# The effect of trauma and PTSD on telomere length: An exploratory study in people exposed to combat trauma

**DOI:** 10.1038/s41598-017-04682-w

**Published:** 2017-06-29

**Authors:** Tae Yong Kim, Se Joo Kim, Jong Rak Choi, Seung-Tae Lee, Jieun Kim, In Sik Hwang, Hae Gyung Chung, Jin Hee Choi, Hae Won Kim, Se Hyun Kim, Jee In Kang

**Affiliations:** 1 Department of Neuropsychiatry, Veterans Health Service Medical Center, Seoul, South Korea; 2 Institute of Behavioral Science in Medicine & Department of Psychiatry, Yonsei University College of Medicine, Seoul, South Korea; 3 Department of Laboratory Medicine, Yonsei University College of Medicine, Seoul, South Korea; 4 Brain Korea 21 PLUS Project for Medical Science, Yonsei University College of Medicine, Seoul, South Korea; 5 Department of Psychiatry and Institute of Clinical Psychopharmacology, Dongguk University Ilsan Hospital, Goyang, Gyeonggi South Korea

## Abstract

Telomere length has been suggested to be a cellular marker for age-related diseases as well as psychosocial stress. The present study investigated whether telomere length is associated with post-traumatic stress disorder (PTSD) among veterans exposed to combat trauma in the Vietnam War. The potentially associated factors on cellular aging were considered. Korean male veterans with (*n* = 122) and without (*n* = 120) PTSD were included and leukocyte telomere length was measured with a quantitative PCR-based technique. As a whole, no significant difference in telomere length was found between PTSD and non-PTSD groups. In linear regression analysis stratified by trauma levels, among veterans exposed to severe combat (*n* = 45), PTSD status (B = −1.176, t = −2.259, *p* = 0.029), antidepressant use (B = 0.168, t = 2.528, *p* = 0.015), and education level (B = 0.019, t = 2.369, *p* = 0.023) affected telomere length. However, among veterans with light-to-moderate combat exposure (*n* = 197), only age (B = −0.007, t = −2.434, p = 0.016) and education level (B = 0.010, t = 2.295, *p* = 0.023) were associated with telomere length. In the *Post-hoc* analysis, antidepressant use was associated with longer telomere length in subjects exposed to severe combat. Our exploratory results suggest that PTSD status in combination with severe trauma may be associated with accelerated telomere shortening, and that antidepressant use may have a protective effect on telomere dynamics.

## Introduction

Telomeres refer to protective non-coding segments of tandem TTAGGG repeats at the ends of chromosomes^1^. Telomeres play a crucial role in preventing chromosome fusion and maintaining genomic stability^2^. They may shorten during repeated cell divisions, aging, and in response to cumulative exposure to oxidative stress or other genotoxic environments^3–5^. Telomere shortening is therefore used as a biomarker of cellular aging and medical mortality^6–8^.

Much attention has been paid to the role of stress in aging over the past two decades. Since the first report of a relationship between perceived stress and shortened telomere length^3^, shortening of telomere length has been suggested to be a promising cellular marker for psychosocial stress^3, 9, 10^. Recent studies showed that accelerated telomere shortening is associated with poorer mental health including depression and anxiety^11^, and trauma exposure^12^. The prospective Dunedin Study showed that persistent internalizing psychiatric disorders predicted telomere shortening after accounting for multiple potential associated factors including childhood maltreatment, smoking, and psychotropic medication use, specifically in males^13^. A recent meta-analysis (n = 14,827) of adult psychiatric disorders and telomere length showed that psychiatric disorders as a whole have a robust effect on telomere shortening (Hedge g = −0.50, *p* < 0.001), compared to healthy controls^14^. Furthermore, a 6-year longitudinal study showed that patients with depressive and anxiety disorders (n = 2,292) had significant telomere shortening even after controlling for lifestyle and health variables, compared to healthy controls^11^.

Psychosocial stress is a well-known risk factor for psychiatric disorders. In particular, post-traumatic stress disorder (PTSD) is among only a few psychiatric diagnoses that uniquely depend on a triggering external traumatic event or stressor^15^. PTSD has been reported to be significantly associated with premature development of age-related diseases including cardiovascular diseases^16, 17^ and cognitive decline^18^. Although the biological mechanisms linking PTSD to excess medical morbidity and mortality are unclear, severe psychosocial stress in PTSD may lead to stress-related oxidative damage to cells and accelerated telomere shortening^19^.

The present study examined whether leukocyte telomere length is influenced by PTSD status among individuals exposed to combat trauma. To better understand the association between PTSD and telomere length, we performed analyses stratified by trauma exposure levels, given that higher trauma exposure confers a higher risk of PTSD^20^. In addition, the influence of potentially associated factors on cellular aging, including demographic factors (age, education, and economic status), alcohol consumption^21^, smoking status^22^, and antidepressant medication^9^ which have been previously reported to be associated with telomere length, were considered for analyses. Our main hypothesis was that PTSD status would be associated with decreased telomere length in individuals exposed to severe trauma.

## Results

Of the 256 people who participated in the interview, data from 14 veterans were not included in the final analysis: four veterans who scored 0 on the self-report CES were excluded, 1 sample was excluded due to insufficient DNA, and another 9 participants were excluded because they had telomere length values that were outliers based on intra-assay CV. Thus, the final analyses included 122 patients with PTSD and 120 control subjects. The mean age of the PTSD group was 62.99 years (standard deviation; SD = 3.40 years), while the mean age of the non-PTSD group was 62.94 years (SD = 4.41 years). No significant between-group differences were observed in demographic characteristics such as age, education level, marital status, or socioeconomic status.

As a whole, no significant difference in relative telomere length was found between PTSD and non-PTSD groups based on Student’s *t*-test (0.64 ± 0.19 vs. 0.64 ± 0.16, *p* = 0.75). Subjects with PTSD were more likely to engage in harmful alcohol drinking based on AUDIT scores, which were 11.59 ± 10.91 in the PTSD group and 6.71 ± 7.63 in the non-PTSD group (*p* < 0.001). In addition, the proportions of non- or light smokers and heavy smokers were 50% (*n* = 61) and 50% (*n* = 61) in the PTSD group versus 39.2% (*n* = 47) and 60.8% (*n* = 73) in the non-PTSD group, respectively (*X* ^*2*^ = 2.873, *p* = 0.09). In the present sample, 71.3% and 20.8% of subjects with and without PTSD had taken psychoactive medications, and the most common medication used was a selective serotonin reuptake inhibitor (SSRI).

Combat exposure was classified as severe (≥25) or light-to-moderate (<25) based on CES score based on a previous study which reported that higher combat exposure confers a higher risk of PTSD, compared to light-to-moderate combat exposure^20^. Among all participants, 81.4% (*n* = 197) reported light-to-moderate combat exposure, and 18.6% (*n* = 45) reported severe combat exposure. No significant difference in relative telomere length was found between subjects exposed to severe trauma and subjects exposed to light-to-moderate trauma based on Student’s *t*-test (0.67 ± 0.20 vs. 0.63 ± 0.17, *p* = 0.30). Among subjects with PTSD, the corresponding proportions of light-to-moderate versus severe levels of combat exposure were 72.1% (*n* = 88) and 27.9% (*n* = 34), respectively. Among subjects without PTSD, the corresponding proportions of light-to-moderate versus severe levels of combat exposure were 90.8% (*n* = 109) and 9.2% (*n* = 11), respectively. The distribution of CES categories between PTSD and non-PTSD groups was significantly different (*χ* ^*2*^ = 13.98, df = 1, *p* < 0.001), with a higher proportion of severe combat exposure in subjects in the PTSD group than subjects in the non-PTSD group. Socio-demographic and clinical characteristics of PTSD and non-PTSD subjects stratified by trauma exposure levels (severe vs. light-to-moderate exposure) are presented in the Table 1, and statistics for comparisons among the four subgroups are also shown. No significant difference in relative telomere length was observed among the four subgroups based on analysis of variance (ANOVA) or analysis of covariance (ANCOVA) with adjustment for socio-demographic variables (*p* = 0.55, adjusted *p* = 0.39).

**Table 1:** Socio-demographic and clinical characteristics of PTSD and non-PTSD subjects stratified by trauma exposure levels.

Characteristics	Severe combat exposure (*n* = 45)		Light-to-moderate combat exposure (*n* = 197)			
	PTSD (*n* = 34)	Non-PTSD (*n* = 11)	PTSD (*n* = 88)	Non-PTSD (*n* = 109)	F or χ^2^	*p*
Age	63.38 ± 3.13	62.82 ± 5.74	62.84 ± 3.50	62.95 ± 4.23	0.16	0.99
Education (years)	10.35 ± 3.28	9.45 ± 4.28	10.38 ± 2.63	10.56 ± 3.03	0.47	0.70
Socioeconomic status: High/Medium/Low, n	7/14/13	2/5/4	18/41/29	18/50/41	1.26	0.98
AUDIT score	13.09 ± 10.77	6.73 ± 7.56	11.01 ± 10.97	6.71 ± 7.67	5.79	0.001
Heavy smoker: Yes/No, n	20/14	8/3	41/47	65/44	4.93	0.17
SSRI use: Yes/No, n	22/12	0/11	53/35	19/90	56.74	<0.001
Relative telomere length	0.66 ± 0.21	0.71 ± 0.16	0.64 ± 0.18	0.63 ± 0.16	0.71	0.55

AUDIT, Alcohol Use Disorders Identification Test; SSRI, selective serotonin reuptake inhibitor.

Linear regression models of telomere length were tested separately according to the two levels of trauma exposure severity. Linear regression analysis with backward selection was performed using relative telomere length (T/S ratio) as the dependent variable and PTSD status, age, education, socioeconomic status, alcohol use, smoking status, and SSRI use as independent variables, based on a priori assumption. Among veterans exposed to severe combat (n = 45), PTSD status, SSRI use, and education level affected telomere length (F = 3.681, *p* = 0.019); these variables explained 21.2% of the total variance (Table 2). Among veterans with light-to-moderate levels of combat exposure (n = 197), only age and education levels were associated with telomere length (F = 5.101, *p* = 0.007) (Table 2).

**Table 2:** Linear regression analysis to predict relative telomere length among combat veterans according to trauma exposure levels.

Significant associated factors		B	S.E.	t	*p*
**Severe combat exposure (** ***n*** ** = 45)**					
**Model;** F = 3.681	**Education level**	0.019	0.008	2.369	0.023
* p* = 0.019	**PTSD status**	−1.176	0.078	−2.259	0.029
R^2^ = 0.212	**SSRI use**	0.168	0.067	2.528	0.015
	Constant	0.526	0.094	5.606	<0.001
**Light-to-moderate exposure (** ***n*** ** = 197)**					
**Model;** F = 5.101	**Education level**	0.010	0.004	2.295	0.023
*p* = 0.007	**Age**	−0.007	0.003	−2.434	0.016
R^2^ = 0.050	Constant	0.997	0.192	5.201	<0.001

**Figure 1:**
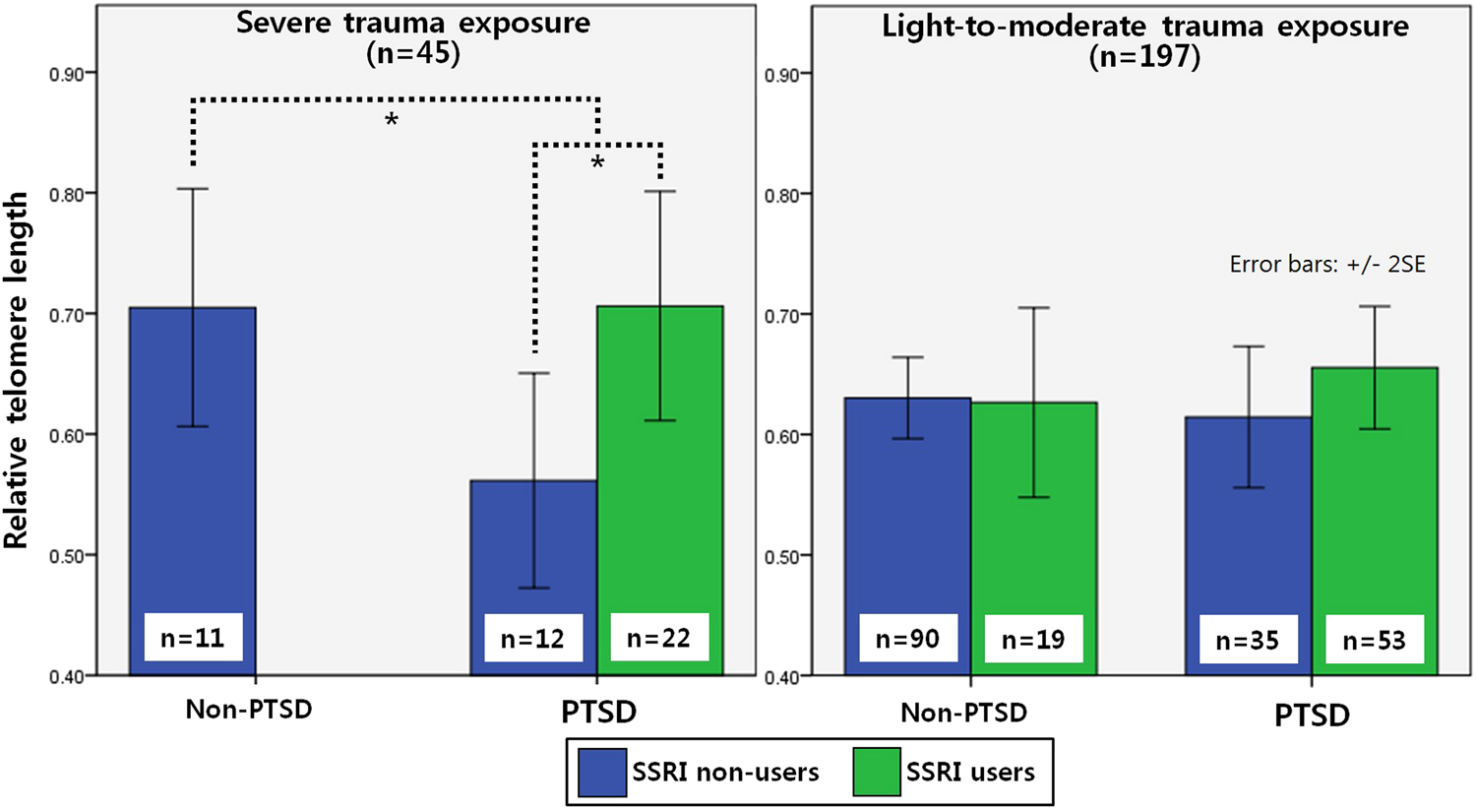
Comparisons of relative telomere length in PTSD and non-PTSD groups of combat veterans according to SSRI use and trauma exposure levels.

In the *Post-hoc* ANCOVA analysis with adjustment for age and education level (fixed factors: PTSD status and SSRI use, a dependent variable: telomere length) stratified by trauma levels, PTSD patients on SSRI (*n* = 22) had significantly higher T/S ratios than patients not on SSRI (*n* = 12) among individuals exposed to severe trauma (*p* = 0.02), while neither PTSD status nor SSRI use were associated with telomere length among individuals exposed to light-to-moderate trauma (Fig. 1).

**Figure 2:**
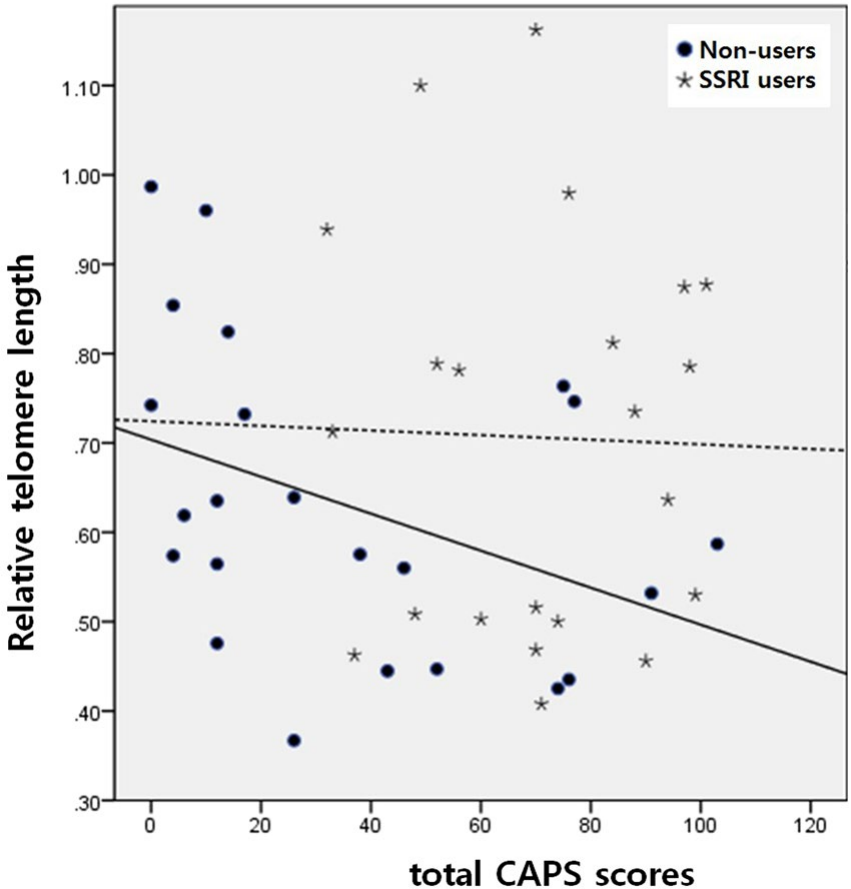
Partial correlation between relative telomere length and PTSD symptom scores within SSRI non-users (*n* = 23) or within SSRI users (*n* = 22) among veterans exposed to a severe level of trauma (*n* = 45).

Furthermore, in partial correlation analysis after adjusting for age and education level, a significant negative correlation between T/S ratio and total CAPS score was found within SSRI non-users (n = 23, *p* = 0.026, *r* = −0.484), while no correlation was found within SSRI users (n = 22, *p* > 0.05) among individuals exposed to severe combat (Fig. 2).

## Discussion

The present study investigated whether leukocyte telomere length is influenced by trauma and PTSD in a relatively homogenous population of older Korean males exposed to the same traumatic event, namely combat in the Vietnam War. We demonstrated that PTSD status and SSRI use affected leukocyte telomere length in people exposed to severe combat trauma. Our results suggest that PTSD status in combination with severe trauma exposure may accelerate cellular aging as reflected by decreased telomere length, and that SSRI treatment may protect against this decrease in telomere length.

Telomere dynamics in humans are affected by various environmental factors and complex pathways throughout our lifespans. Among the veterans evaluated in this study, while no significant difference was observed in T/S ratios between PTSD and non-PTSD groups, several significant factors, including PTSD status, were found after adjusting for other factors according to level of trauma exposure severity. The finding of shorter leukocyte telomere length in PTSD patients exposed to severe trauma is consistent with the growing evidence that stress affects telomere length^19, 23^. Chronic stress, such as a high perceived stress level, has been reported to be associated with telomere length and health effects^24^. A recent comprehensive review of PTSD and senescence reported that PTSD was associated with accelerated aging and higher medical morbidity and mortality due to age-related diseases^25^. These findings suggest that the decrease in telomere length (and the inferred acceleration in cellular aging) associated with severe stress may be a promising biomarker of psychosocial stress as well as the progression of age-related diseases or mortality.

However, it is not clear from our results whether telomere length is a long-lasting molecular signature due to severe trauma exposure or a molecular signature resulting from a psychiatric illness, namely PTSD. Furthermore, significant debate exists over the impact of PTSD development on cellular aging. Unlike our study in which decreased telomere length was associated with PTSD status after severe trauma, a recent longitudinal study of Dutch soldiers (n = 96, before and 6 months after deployment in Afghanistan) showed that increased telomere length was significantly associated with development of PTSD symptoms^26^. In our analyses stratified by trauma levels, telomere length was not associated with PTSD status in veterans with light-to-moderate levels of combat exposure. Although it is difficult to directly compare the changes in young soldiers over a 6 month period with our cross-sectional findings in old veterans tested several decades after combat exposure, accelerated cellular aging seems to have a complex relationship with PTSD symptom and be differentially influenced by potential factors including levels of trauma exposure and chronicity/severity of PTSD. Since high stress levels can significantly affect the duration and symptom severity of PTSD, PTSD status after severe trauma exposure may exert a greater influence on telomere length than after light trauma. It is also possible that the severity of trauma exposure may serve as a moderator between PTSD and accelerated cellular aging. To verify the effects of trauma exposure and PTSD development on telomere dynamics, further multi-wave longitudinal studies are warranted. In addition, to examine the impacts of trauma exposure itself on telomere length, it would be helpful to include as comparison groups both trauma-unexposed healthy controls and trauma-exposed non-PTSD subjects.

Importantly, our result demonstrated that SSRI use was significantly associated with telomere length; SSRI users had relatively greater telomere length ratios than SSRI non-users among those veterans with severe trauma. In addition, non-users of SSRI had a significant negative association between telomere length and PTSD symptom severity, while this negative association was not present in SSRI users. Similarly, a previous study of major depression reported a significant negative association between lifetime exposure to untreated depression and telomere length, while this negative association was not present in subjects receiving antidepressants^9^. These differential findings in antidepressant users and non-users suggest that active SSRI treatment for PTSD recovery may mitigate the potentially harmful effects of PTSD on telomere length in people exposed to traumatic stress. Although the cross-sectional nature of our study precludes determining causal relationships between SSRI use and telomere length in subjects with PTSD, SSRI treatment may exert a protective effect on accelerated cellular aging via reversing the effects of chronic PTSD on telomere length. A recent prospective study of 27 depressed patients found an association between telomere length and SSRI response, and suggested that reduced pre-treatment telomere length may be a predictive biomarker of poor treatment responses to SSRI in major depression^27^. Further prospective studies with multi-wave longitudinal data examining the effects of SSRI interventions on telomere length in people exposed to severe trauma might shed light on the role of SSRIs and their pathophysiological mechanisms in PTSD and telomere dynamics.

The question of how PTSD impacts cellular aging remains unclear. Maladaptive autonomic regulation in PTSD may be a key mechanism that accelerates cellular aging and mortality. Patients with PTSD have abnormal autonomic oscillations related to hyperarousal symptoms even in response to ordinary situations, and re-experience traumatic events, which may lead to severe psychosocial distress and dysfunctional lifestyles^28^. In addition, as Miller *et al*. proposed, chronic and repeated HPA-axis activation may result in glucocorticoid-related oxidative damage to neurons and, in turn, PTSD-related cellular aging^29^. Another possible biological mechanism linking PTSD and accelerated cellular aging may be chronic inflammatory processes that promote oxidative stress. Altered immune system function, including excess inflammation and high levels of cytokines, has been implicated in chronic PTSD among traumatized individuals^30–32^. Taken together, alterations in the autonomic nervous system, the HPA axis, and/or inflammatory responses to severe stress may contribute to oxidative stress and accelerated cellular aging^19^. The underlying pathophysiology of PTSD and the link between this and cellular aging require additional research.

We also found that education level was significantly associated with leukocyte telomere length. Findings regarding an association between education level and telomere length have been inconsistent across studies. While several previous studies reported a significant relationship between shorter telomere length and lower educational attainment^33–35^, similar to our finding, some observed no relationship between telomere length and education^6, 36^. Education level may be a proxy risk factor for other predictors of accelerated cellular aging and poor health, because individuals with lower educational attainment may have greater stress exposure, poorer coping strategies for stress regulation, and dysfunctional life-styles^37, 38^.

The present study has several potential limitations. First, the subjects in our study consisted of older male veterans (average age 63.0 years (SD = 3.9 years)) exposed to combat trauma approximately 45 years prior. Although the relatively narrow age variation of our subjects may be considered strength, present telomere length is likely to have been influenced by lifetime cumulative burden including various environmental factors over long periods after combat exposure. Second, we did not consider subject-level risk factors, such as genetic polymorphisms and early-life adversity, which can affect adult telomere dynamics. In particular, recent studies have shown that exposure to early-life adversity may be associated with shortened telomere length, which may then contribute to PTSD development and morbidity in later life^39, 40^. Furthermore, we did not consider the effects of some possible confounding factors on telomere length, such as body mass index, amount of physical activity and dietary intake of anti-oxidant foods. Third, we used only qPCR among multiple methods for assessing telomere length. Combined use of qPCR with other sensitive measurements of telomere length such as flow-FISH may aid in detection of subtle differences in telomere length in a clinical setting. Finally, our sample consisted of a relatively small number of individuals with a severe level of trauma exposure, which may lead to a risk of overfitting in the regression model. In addition, multiple testing in the study may potentially increase Type I errors. Despite these limitations, we provided evidence of an association among PTSD, severe trauma, and telomere length, indicating that mental health and psychosocial stress can affect telomere length.

In summary, the presence of PTSD and SSRI use affected leukocyte telomere length in combat veterans exposed to severe combat trauma. Our results suggest that PTSD status in combination with severe trauma may be associated with accelerated telomere shortening, and that SSRI use may have a protective effect on telomere dynamics. Further prospective research in independent PTSD samples with consideration of potential confounding variables is required to clarify how PTSD development, trauma level, and interventions such as SSRI use are interrelated with telomere length.

## Methods

### Participants

All subjects gave their written informed consent prior to participation. A total of 256 male veterans who were on active duty during the Vietnam War (from 1965 to 1973) were recruited through advertisements at the Veterans Health Service (VHS) Medical Center and interviewed based on DSM*-*IV*-*TR^15^ by a trained psychiatrist. Exclusion criteria for the study were a history of head trauma, organic brain syndrome including cerebrovascular accidents or dementia, psychosis or bipolar disorder, or dependence on substances other than alcohol and nicotine. This study was approved by the institutional review board of the VHS Medical Center, Seoul, Korea. The methods were performed in accordance with relevant guidelines and regulations.

### Assessment of clinical characteristics

The Clinician-Administered PTSD Scale (CAPS), which is one of the most useful measures of PTSD^41, 42^, was applied to assess the presence of PTSD and the frequency and intensity of each PTSD symptom dimension. PTSD status was determined by weighing both the symptom frequency and intensity based on the liberal scoring rule^43^; frequency ≥1 (occurred at least once during the time frame) and intensity ≥2 (at least moderately distressing) for each item.

In addition, the Combat Exposure Scale (CES) was used to assess the level of combat trauma experienced by veterans^44^. The CES, a widely used 7-item, 5-point Likert self-rating scale, has demonstrated good internal consistency and reliability in previous research of combat-related PTSD^44^. Based on the CES results, subjects were classified dichotomously as having been exposed to light-to-moderate levels (<25) versus severe levels of combat exposure (≥25)^20^.

The Alcohol Use Disorders Identification Test (AUDIT) was used to identify participants with problematic alcohol use^45^. In addition, long-term smoking exposure was determined by multiplying the number of years smoked by the number of packs smoked per day. Then, using a cutoff of 10 pack-years, subjects were classified into two groups: non- or light smokers (<10 pack-years) and heavy smokers (≥10 pack-years).

### Telomere length measurement

Genomic DNA was prepared from peripheral blood samples using a nucleic acid isolation device, the QuickGene-mini80 (FUJIFILM, Tokyo, Japan). Samples were normalized in 96-well microtiter plates to a DNA concentration of 20 ng. A standard sample at 100-ng/μL was used to create a DNA standard of 80 ng/μL, which was then serially diluted to produce standards of 40-ng/μL, 20-ng/μL, 10-ng/μL, 5-ng/μL, 2.5-ng/μL, and 1.25-ng/μL. Leukocyte telomere length was measured using the real-time quantitative polymerase chain reaction (qPCR) assay developed by Cawthon^46^. The primers (5′ → 3′) used for the telomere and the single copy gene (SCG) amplification were as follows: telg (0.9 μL, 10 μM): ACACTAAGGTTTGGGTTTGGGTTTGGGTTTGGGTTAGTGT, telc (0.9 μL, 10 μM): TGTTAGGTATCCCTATCCCTATCCCTATCCCTATCCCTAACA, SCG beta-globin primer hbgu (0.5 μL, 10 μM): CGGCGGCGGGCGGCGCGGGCTGGGCGGcttcatccacgttcaccttg, SCG beta-globin primer hbgd (0.5 μL, 10 μM): GCCCGGCCCGCCGCGCCCGTCCCGCCG gaggagaagtctgccgtt. The thermal cycling profile for the telomere PCR was as follows: 95 °C for 15 min, followed by 2 cycles of 94 °C for 15 s and 52 °C for 15 s, 32 cycles of 94 °C for 15 s, 62 °C for 10 s and 74 °C for 15 s with fluorescent signal acquisition, 84 °C for 10 s, and finally 88 °C for 15 s with signal acquisition. For both the telomere PCR and the SCG PCR, the final reaction volume (10 μl) consisted of 2× SYBR Green PCR Master Mix (Bioline Ltd, UK), 100 ng of template, and the respective primers. For internal quality control, DNA samples from all participants were simultaneously tested in triplicate in two runs. The intra-assay mean value range of the coefficient of variation (CV) from the results of the different DNA sample assays was 0.3–22.3%. The average intra-assay CV for the relative telomere to single copy gene (T/S) ratio within triplicates was 4.34% after outlier removal.

### Statistical analyses

Socio-demographic and clinical characteristics were compared between groups using Student’s *t*-test, ANOVA, or ANCOVA. For categorical variables, group comparisons were performed using the chi-square or Fisher’s exact tests when the expected cell counts are small. Partial correlation analysis was conducted to examine relationships between PTSD symptom scores and relative telomere length to adjust for possible confounders. Linear regression analysis was used to identify major determinants that best predicted telomere length. In addition to PTSD status and trauma exposure levels, when statistical significance was found in the Pearson’s correlation analyses or when a factor was reported to be a predictor of telomere length in previous studies, these factors were also considered to be independent variables in the regression models. Thus, the effects of PTSD status and clinically important variables including age, education, socioeconomic status, alcohol use (AUDIT score), smoking status (heavy smokers vs. non- or light smokers), and SSRI use (user vs. non-user) on relative telomere length (T/S ratio) were tested using linear regression analysis with backward selection model. Statistical analyses were performed using the Statistical Package for the Social Sciences (SPSS Inc., Chicago, IL, USA) version 23.0. Significance level was accepted at *p* < 0.05, and all tests were two-tailed.
